# Choosing a Benchtop Sequencing Machine to Characterise *Helicobacter pylori* Genomes

**DOI:** 10.1371/journal.pone.0067539

**Published:** 2013-06-28

**Authors:** Timothy T. Perkins, Chin Yen Tay, Fanny Thirriot, Barry Marshall

**Affiliations:** Pathology and Laboratory Medicine, The University of Western Australia, Perth, Western Australia, Australia; J. Craig Venter Institute, United States of America

## Abstract

The fully annotated genome sequence of the European strain, 26695 was first published in 1997 and, in 1999, it was directly compared to the USA isolate J99, promoting two standard laboratory isolates for *Helicobacter pylori* (*H. pylori*) research. With the genomic scaffolds available from these important genomes and the advent of benchtop high-throughput sequencing technology, a bacterial genome can now be sequenced within a few days. We sequenced and analysed strains J99 and 26695 using the benchtop-sequencing machines Ion Torrent PGM and the Illumina MiSeq Nextera and Nextera XT methodologies. Using publically available algorithms, we analysed the raw data and interrogated both genomes by mapping the data and by *de novo* assembly. We compared the accuracy of the coding sequence assemblies to the originally published sequences. With the Ion Torrent PGM, we found an inherently high-error rate in the raw sequence data. Using the Illumina MiSeq, we found significantly more non-covered nucleotides when using the less expensive Illumina Nextera XT compared with the Illumina Nextera library creation method. We found the most accurate *de novo* assemblies using the Nextera technology, however, extracting an accurate multi-locus sequence type was inconsistent compared to the Ion Torrent PGM. We found the *cag*PAI failed to assemble onto a single contig in all technologies but was more accurate using the Nextera. Our results indicate the Illumina MiSeq Nextera method is the most accurate for *de novo* whole genome sequencing of *H. pylori.*

## Introduction

*Helicobacter pylori* is an important human pathogen, infecting more than 50% of the world’s population [1]. It is micro-aerophilic, flagellated and gram-negative and is generally transmitted vertically from mother to child in the early stages of life, colonising and persisting in the gastric mucosa unless treated. Its ability to survive the hostile milieu of the stomach induces a broad spectrum of disease outcomes ranging from chronic gastritis and peptic ulcer disease to gastric cancer or mucosal associated lymphoma [2].

Two unrelated genome sequences were published in 1997 (26695) and 1999 (J99), detailing two similar, compact and low GC genomes [3], [4]. These genomes have become standard laboratory reference genomes. Approximately 6–7% of genes were unique to each strain (most of which were encoded on a hypervariable region) but the overall genomic organisation and predicted proteomes were similar, despite the expectation of high allelic diversity [4].

High-throughput sequencing methodologies generate gigabases of short-read sequence data in a relatively short period of time [5], [6]. Benchtop DNA sequencing machines can produce datasets for as little as USD0.50/Mb [7], with historical prices decreasing faster than Moore’s Law. Two of the most inexpensive benchtop machines are the Illumina MiSeq Personal Sequencer and the Ion Torrent PGM. The Illumina MiSeq employs reversible terminator sequencing by synthesis that incorporates fluorescently labeled dNTPs. Each cycle represents a single base addition to the DNA strand, which is subsequently excited by laser and imaged to determine the incorporated dNTP prior to its subsequent cleavage and the addition of an unlabeled dNTP. The Ion Torrent PGM measures the incorporation of dNTPs using a semiconductor, which serve as miniature pH meters. During the addition of dNTPs, a proton is released, altering the pH of the solution, which is measured by the semiconductor chip. The target DNA is amplified by sequential flooding of dNTPs and the change in pH is relative to the number of incorporated nucleotides. However, homopolymeric tracts are difficult to accurately decipher using this method due to smaller increases in the pH difference with every identical base.

Specialised algorithms are required to interrogate the large datasets and the read lengths are increasing, now commonly greater than 150 bps. The sequence data can be analysed in its raw format, mapped to a reference genome, or assembled *de novo.* As a measure of quality control the raw data can be analysed by the number of *k*-mers, or words with DNA sequence of length equal to *k*. The sequenced genome encodes a finite number of *k*-mers, however, with imprecise sequencing, greater depth will increase the number of *k*-mers present in the output data. The frequency of accurate *k*-mers in the raw dataset is expected to be a function of sequencing depth, with unique *k*-mers expected to be erroneous. Thus, if imprecise technology is used to repeatedly sequence the same *k-*mer (ie sequence to a greater “depth”) then more different *k-*mers will be identified due to the injection of errors. To fully exploit the mapping method, a highly similar reference is required as a scaffold and therefore only small differences can be measured. This methodology is limited if regions of high-genomic variability exist (due to non-mapping of reads), or if regions of DNA are commonly acquired and lost. *De novo* assembly iteratively rebuilds contigs of DNA by matching overlapping *k*-mers across all reads [8]. The number of contigs attained using this method can vary greatly and is highly dependent on the repetitive nature of the genome; the accuracy of the sequence data and length of each read.

Despite the similarity of J99 and 26695, high allelic diversity is evident in the species *H. pylori* from the classically obtained *H. pylori Pub*MLST (public multi-locus sequence typing) database. MLST is a method developed to infer strain-to-strain relationships by analysing the sequences of 7 “house-keeping” genes [9]. The *H. pylori Pub*MLST database houses information on more than 1500 strains isolated world-wide with each gene represented by approximately 1200 different alleles [9]. This massive diversity present within a single species required the use of the program STRUCTURE to be used to group together strains using MLST data [10]. As well as the designation of a sequence type (which is generally unique), the STRUCTURE algorithm assigns a broader group notation to similar strains. Indeed, each group is representative of its point of geographic isolation or migrational heredity and this information has been used to trace pre-historic migrational patterns of the human host [10], [11]. In addition to the 7 “house-keeping” genes, the sequence of the virulence-associated gene encoding for vacuolating cytotoxin A, *vacA,* is also commonly included in analysing strain-to-strain relationships of *H. pylori* [9].

Finally, if present, the Cag pathogenicity island (*cag*PAI) is of major relevance to *H. pylori* clinical disease outcome. The *cag*PAI encodes the protein machinery to express a type 4-secretion system capable of delivering the bacterial onco-protein CagA [12]. CagA is translocated into epithelial cells and is a potent inducer of the pro-inflammatory cytokine, IL-8 [13]. Its genetic variation has recently been analysed by classical sequencing of the entire island of 38 phylo-geographically diverse strains [14]. This is a third area where high-throughput sequencing could simplify analysis of disease causing isolates.

Due to the accessibility of such benchtop sequencing machines and the ability to sequence a whole genome at a lower cost than classical MLST typing, we set out to determine which machine is most appropriate to determine the phylogeny of an *H. pylori* strain. We analysed the sequence and assembly fidelity by re-sequencing two published *H. pylori* strains (J99 and 26695). We chose to analyse the accuracy of all coding sequences, the accuracy of reconstructing the MLST and each technology’s ability to correctly assemble the clinically important *cag*PAI.

Our data show that by analysing the 31-mer compositions of the raw data, there is an inherently higher error rate in the Ion Torrent PGM sequence data than MiSeq sequencing. For Illumina MiSeq, the Nextera library preparation kit is the preferable approach than the Nextera XT kit. Peculiarly, our raw data contain 31-mers that are unique in the dataset that map to the reference genome but have different GC compositions for different methods. Our data show that Ion Torrent and Illumina Nextera are adequate technologies for mapping strategies, yet the Nextera XT had a significantly higher number of non-covered nucleotides. Our data show that the Illumina Nextera system is the best method for *de novo* assembly, yet, the absence of the *atpA* MLST allele in one assembly reduces its ability to confidently infer phylogeographic relationships by whole genome sequencing and subsequent *de novo* assembly in *H. pylori*.

## Materials and Methods

### Bacterial Growth and Genomic DNA Extraction

Bacteria were grown as previously described by Tay *et al* [15]. Briefly, *H. pylori* were cultured on Columbia blood agar plates (CBA) (Columbia agar base; Oxoid, Adelaide, Australia) with 5% horse blood and incubated at 37°C and 10% CO2 for 48 hours before DNA extraction. The genomic DNA was extracted using the DNeasy Blood & Tissue Kit (Qiagen, Valencia, CA) according to the manufacturer's protocol. Briefly, the cells were harvested in saline and centrifuged at 13000 rpm for 1 minute. The pellet was resuspended in 180 µl ATL Buffer. After the addition of 20 µl Proteinase K (20 mg/ml) the mixture was incubated for 1 hour at 56°C. Then, mixed thoroughly with 200 µl of AL Buffer, followed by the addition of 200 µl of 95% ethanol to the lysate. The genomic DNA was washed in the DNeasy Mini spin column according to the manufacturer's instructions and eluted from the column after 1 min incubation with 100 µl of TE Buffer.

### Ion Torrent Sequencing

100 ng of *H. pylori* genomic DNA was sheared to approximately 200–300 bp using an S2 sonicator (Covaris, UWA). Barcoded libraries were prepared using an Ion Xpress Fragment Library kit (Life Technologies, USA). Size selection (insert sizes 200–250 bp) was performed by gel excision (E-gel, Invitrogen) and the libraries were assessed and quantified using a Bioanalyser 2100 (Agilent Technologies, USA). Individual libraries were then diluted to 9 pM for template preparation using a OneTouch Template 200 kit (Life Technologies, USA) and enriched. Sequencing was performed on a PGM (Ion Torrent) using 520 flows (generating circa 200–250 bp read lengths) on a 316 sequencing chip. After sequencing, signal processing and basecalling was performed using TorrentSuite 1.5.

### Illumina Library Preparation and Sequencing

Preparation of Nextera libraries was performed with 50 ng of genomic DNA according to the Nextera protocol (Ver. October 2011). Briefly, DNA was fragmented using 5 µl of Tagment DNA enzyme with 20 µl of Tagment DNA buffer (Illumina Inc., San Diego, CA). Tagmentation reactions were performed by incubation at 55°C for 5 min followed by purification of the tagmented DNA by the use of the Zymo Clean and Concentrator-5 kit (Zymo Research, Orange, CA). Purified DNA was eluted from the column with 25 µl of resuspension buffer. Purified tagmented DNA (20 µl) was used as the template in a 50 µl limited-cycle PCR (5 cycles) and processed according to the Nextera protocol. Amplified DNA was purified using 30 µl AMPure XP beads (Beckman Coulter Inc, Australia.). The fragment size distribution of the tagmented DNA was analysed utilising a 2100 Bioanalyser with a High Sensitivity DNA assay kit (Agilent Technologies, Santa Clara, CA). DNA libraries were normalised to 2 nM, pooled in equal volumes and then denatured with 0.2 N NaOH according to the Nextera protocol. Preparation of Nextera XT libraries was performed with 1 ng of genomic DNA according to the Nextera XT protocol (Ver. May 2012). Briefly, the DNA was fragmented in 5 µl of Amplicon Tagment Mix and 10 µl of Tagment DNA buffer (Illumina, San Diego, CA, USA). Tagmentation reactions were performed by incubation at 55°C for 5 min followed by neutralisation with 5 µl of Neutralise Tagment Buffer for 5 min. Tagmented DNA (25 µl) was used as the template in a 50 µl limited-cycle PCR (12 cycles) and processed as outlined in the Nextera XT protocol. Amplified DNA was purified using 90 µl of AMPure XP beads then normalised with 45 µl of combined Library Normalisation beads/additives. In preparation for cluster generation and sequencing, equal volumes of normalised library were combined, diluted in hybridisation buffer and heat denatured. Libraries were sequenced using the MiSeq Personal Sequencer (Illumina Inc., San Diego, CA, USA) running version MiSeq Control Software Version 1.1.1.

### Data Analysis

The raw sequence data was analysed using the R package qrqc [16]. The 31-mers were counted and analysed using the package meryl [17]. The GC content of 31 mers was determined using the script geecee from the EMBOSS package [18]. Significant differences between the population of unique 31 mers and frequently occurring 31 mers was assessed using the R-script for Student’s t-test. All data were mapped to a reference genome using the algorithm BWA [19]. Illumina data were mapped using the default settings and the Ion Torrent data were mapped using the BWA optimised tmap script [20]. The data were assembled using different algorithms. Illumina data were assembled using Velvet assembler with a *k-*mer set to 71 and automatic coverage cutoff enabled [8]. Ion Torrent data were assembled using Mira assembler (parameters –job = denovo,genome,accurate,iontor IONTOR_SETTINGS -ASSEMBLY:mrpc = 100) [21].

### Accession Codes

All sequences have been deposited to the NCBI Sequence Read Archive (http://www.ncbi.nlm.nih.gov/sra) under the study number SRA065843. SRA sample numbers are SRS388100-SRS388109.

## Results

### Analysis of Raw Sequencing Data Indicates Adequate Sequencing Depth is Achieved by All Methodologies

**Figure 1:**
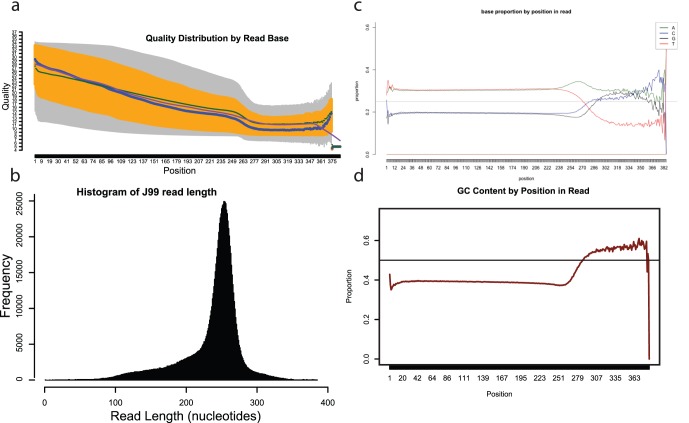
QRQC analysis of J99 raw sequence data derived by Ion Torrent. (a) Plot of the quality distribution by base position. (b) Histogram of the read lengths. (c) Proportions of each base with respect to the position in the read. (d) Mean GC proportion for each position in the reads.

Prior to analysis of data mapped to the reference genome or analysis of *de novo* assemblies, we analysed the raw sequence data to determine the overall quality of the Ion Torrent data. We summarised the raw fastq files (Table 1) and analysed them using qrqc [16] (Figure 1 for strain J99 and Figure S1 for strain 26695). Figure 1 shows that most reads should be trimmed at position 250 as the estimated quality is lower and the nucleotide composition becomes random. We determined the total number of nucleotides in the dataset to calculate the theoretical genome coverage of 153-fold (J99) and 167-fold (26695). The Ion Torrent 200 bp chemistry determined 1.06 million reads, with a median length of 249 nucleotides per read, therefore, adequate representation of each genome was expected (Table 1 and Figure 1b, Figure S1b). Analysis of the estimated accuracy of the entire dataset also suggested that the longer the read, the poorer the quality of sequence data due to the fact that as the length of each read increased, the G+C composition was not congruent with the expected G+C proportion of 0.39 (Figure 1c and d, Figure S1c and d).

**Table 1:** Analysis of raw sequencing data.

	Ion Torrent	MiSeq	
		Nextera	Nextera XT
**J99 (Chromosome length = 1,643,831** **bp; GC composition = 0.39; Number of 31-mers in sequence = 1,643,802)**			
Read lengths	Median = 249; IQ range = 226–260	2×150 bp	2×150 bp
Number of reads	1,061,205	370,146 (paired)	356,151 (paired)
Number of nucleotides	250,830,256	101,903,666	75,623,283
Theoretical coverage	153x	62x	46x
31-mers	219,010,362	79,730,276	54,331,353
Nucleotides/31-mer	0.8731	0.7824	0.7184
Fold more 31-mers than expected	133	48	46
Number of contigs	90	62	673
**26695 (Chromosome length = 1,667,867** **bp; GC composition = 0.39; Number of 31-mers in sequence = 1,667,838)**			
Read lengths	Median = 248; IQ Range 223–259	2x150 bp	2x150 bp
Number of reads	1,197,403	560,078 (paired)	366,121(paired)
Number of nucleotides	281,810,051	127,932,955	76,421,393
Theoretical coverage	167x	77x	46x
31-mers	245,902,472	94,379,396	54,532,193
31-mer/Nucleotides	0.8726	0.7377	0.7136
Fold more 31-mers than expected	169	56	46
Number of contigs	204	38	555

As a means of direct comparison, we sequenced the same aliquot of gDNA using the Illumina MiSeq Nextera sequencing chemistry. There are two available library preparation methods for preparation of gDNA libraries, Nextera and Nextera XT, which require inputs of 50 ng and 1 ng of gDNA, respectively. Both use an enzymatic fragmentation method. The qrqc analysis indicates that the majority of reads are 150 nucleotides and paired, with the estimated nucleotide coverage ranging from 46-fold to 77-fold (Table 1, Figure S2 (J99 Nextera) and Figure S3 (J99 Nextera XT).).

### Analysis of 31-mer Quantities Indicates a Significantly Higher Error Rate in the Ion Torrent Sequence Data

As a measure of quality control, we determined the total number of 31-mers present in the sequencing data and related this to the total number of nucleotides of sequence data. This analysis provides an indication of number of errors in the fastq data, as there are a limited number of possible 31-mers in the reference sequence. Given adequate depth, unique 31-mers can be assumed to be errors and are therefore unlikely to map to the reference genome.

**Figure 2:**
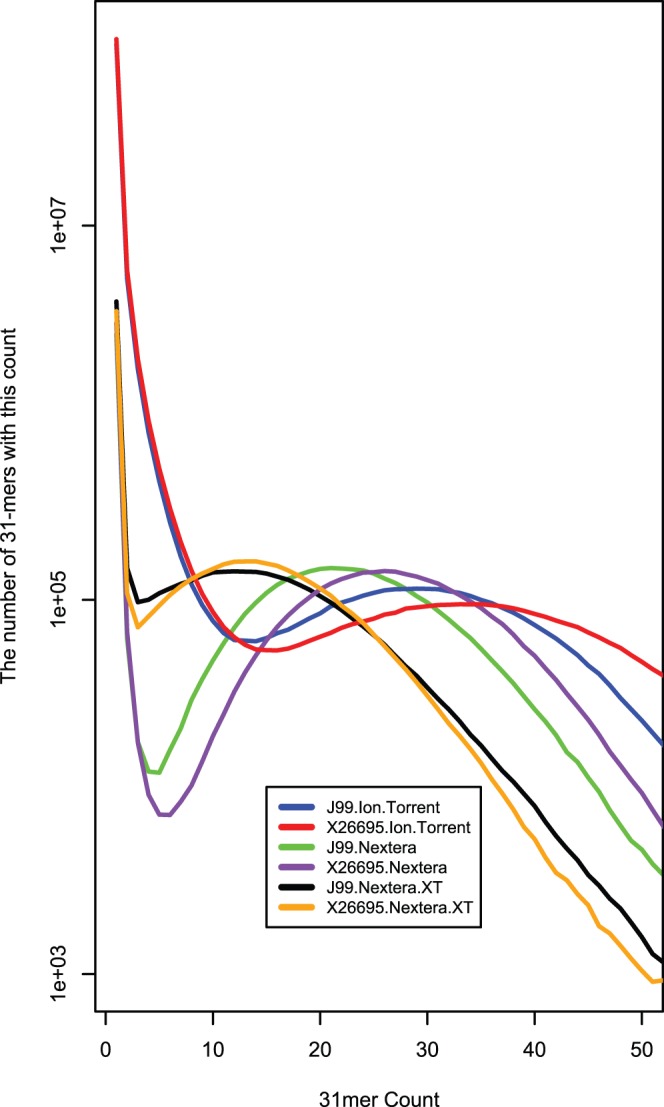
Plot of the frequency of each 31-mer. We determined every possible 31 mer in the sequence data output and then analysed the frequency at which each of those 31-mers occurred. Unique 31-mers are considered sequencing errors given adequate depth. The natural cut-off for each sequencing technology occurs at the turning point in each plot.

**Figure 3:**
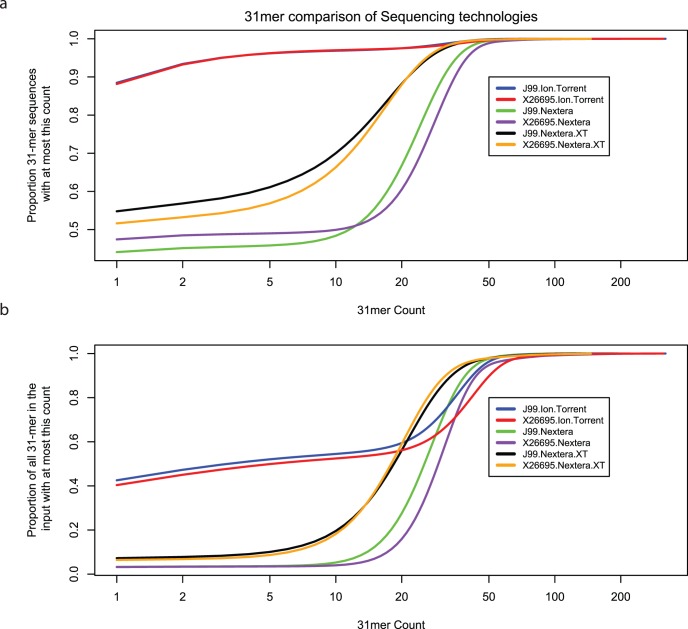
31-mer quality control. Cumulative Proportion of 31-mers with respect to increasing frequency. (a) The cumulative fraction of 31-mers with at most this count. Each 31-mer is treated as a mathematical set and will be listed once. (b) Cumulative fraction of 31-mers with at most this count, ie a 31-mer occurring 5 times will be in the dataset 5 times.

The ratio of 31-mers per nucleotide of sequence data in the Ion Torrent data was greater than those for both Nextera and Nextera XT chemistries (see Table 1). A histogram of the frequency of 31-mer occurrences gives an estimation of the number of errors with respect to the expected coverage for both J99 and 26695 (Figure 2). The theoretical depth is enough for us to expect that all correctly sequenced 31-mers should occur multiple times in the data output, therefore, we plotted the fraction of all 31-mer sequences with respect to the count (Figure 3a). Despite the greater theoretical depth, the Ion Torrent data output has an extremely high proportion of unique 31-mers (J99, 0.8846; 26695, 0.8816) when compared to the Illumina MiSeq Nextera (J99, 0.4410; 26695, 0.4743) and Nextera XT (J99, 0.5479; 26695, 0.5163) indicating that there is an inherently higher error rate. We also analysed the fraction of 31-mers in the input with at most this count, ie a 31-mer with count of 20 will be listed 20 times (Figure 3b).

### Unique 31-mers in the Data Output are Present in the Reference Genomes and have Different GC Content for Each Technology

Unique 31-mers in the output sequence data are expected to be errors given there is adequate depth to cover each genome more than 50-times. To determine if these mapped unique 31-mers were similar in GC content to highly covered regions, we compared their GC content to 10^5^, randomly chosen, highly frequent 31-mers (occurring at a frequency between 20 and 50). This analysis may provide insights as to the reason for the low coverage. We found significant differences in GC content across all sequencing technologies and library preparation methods (Figure S4 and Table 2). The GC content of the Ion Torrent mapped unique reads was significantly higher for both genomes, suggesting a technical reason for the lack of coverage. Interestingly, the GC content of unique 31-mers that map to the reference genomes derived by the Illumina library preparation methods (Nextera and Nextera XT) identified conflicting results. The GC content of unique, mapped 31-mers was significantly different to the GC content of highly frequent 31-mers in both methods. However, the average GC content of unique and mapped 31-mers using the Nextera XT method was lower than expected and using Nextera it was was higher (Table 2, Figure S4b and c). These data also suggest there is a technical reason for the lower coverage obtained for these sequences. Furthermore, the number of mapped unique 31-mers in the Nextera XT dataset is 10-fold higher than the Nextera XT dataset, which is likely due to the reduced overall coverage. The proportion of mapped unique 31-mers of the total number of unique 31-mers is relatively small for all technologies except the Nextera XT (Table 2).

**Table 2:** Analysis of unique 31-mers that map to the references.

Ion Torrent	Analysis of mapped unique 31-mers		Mean GC content of 31-mers		
Sample	Count of mapped unique 31-mers (total unique 31 mers in dataset)	Percentage of mapped unique 31-mers	Mapped unique 31-mers	Highly frequent 31-mers	P-value
J99	9211 (93154944)	0.0099%	0.440	0.362	2.2e-16
26695	9092 (99226294)	0.0092%	0.411	0.357	2.2e-16
MiSeq (Nextera)					
J99	3869 (2618591)	0.148%	0.397	0.351	2.2e-16
26695	2685 (3014441)	0.089%	0.396	0.353	2.2e-16
MiSeq (Nextera XT)					
J99	55035 (3929373)	1.40%	0.280	0.407	2.2e-16
26695	34942 (3487475)	1.00%	0.277	0.402	2.2e-16

Bracketed number represents the total number of unique 31-mers in the dataset.

### The BWA Algorithm does not Introduce Mapping Artifacts for J99 or 26695

Prior to analysing the experimentally derived sequencing data, we simulated a dataset and mapped the data to the reference genome to determine the fidelity of the bwa mapping algorithm [19] and to identify any false positive variants. We simulated a dataset of Illumina-like reads from each reference genome of 150 bp, paired-ended using wgsim [22] with no errors and set the parameters based on the data derived by each Illumina sequencing method (Table S1 in File S1). The data were mapped and variants called. Similarly, the consensus sequence derived from and mapped to J99, identified no false-positive base-pair differences. We noted that the published 26695-reference sequence is not finished precisely and contains regions of undetermined bases (detailed in Table S2 in File S1). As expected, the undetermined bases in the 26695 reference were the only bases where variants were identified by the simulated dataset, indicating that the bwa algorithm is appropriate for further mapping analyses.

### Nextera XT Coverage is Significantly Lower than Nextera when the Data are Mapped

We mapped the data derived by Nextera and Nextera XT sequencing to the J99 genome sequence using the bwa algorithm to determine the precision and accuracy of the sequencing technologies. The insert sizes and standard deviations derived by the analysis of paired reads are detailed in Table S1 in File S1 (these parameters were used to simulate the Illumina-like sequence data).

**Figure 4:**
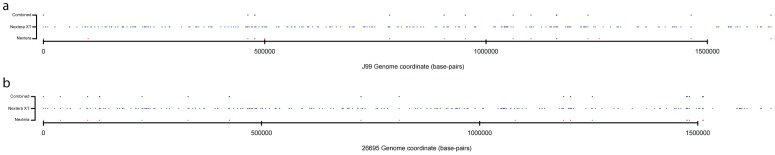
Regions with inadequate sequence depth. Regions in J99 (a) and 26695 (b) where the depth is less than or equal to 3 nucleotides of coverage for Nextera (red), Nextera XT (blue), with black representing common non-covered bases.

The average nucleotide coverage afforded by each technology in these experiments was 62-fold (Nextera) and 46-fold (Nextera XT). Reference nucleotides with 3 or fewer mappings were regarded as non-covered bases. Despite adequate estimated depth, there were regions of the J99 genome within which sequence data did not map (Figure 4a and Table S3 in File S1). The number of nucleotides (nc) with no sequence data mapped increased significantly from 941 nc using Nextera to 12,508 nc using Nextera XT technology, despite a similar number of sequence reads and expected depth of coverage. Of these non-covered regions, 784 nucleotides were common across the technologies.

We mapped the data derived by both Illumina methods from strain 26695 to the published 26695-genome sequence (for insert sizes determined by mapping paired reads see Table S1 in File S1). Expected coverages were 77-fold and 46-fold for Nextera and Nextera XT, respectively and non-covered nucleotides were more abundant for the Nextera XT (10,730 nc) compared to the Nextera (699 nc) library preparation method, with 624 nc common across the two methods (Table S4 in File S1).

### Nextera Mappings Identify Differences in the Template DNA for both J99 and 26695

**Figure 5:**
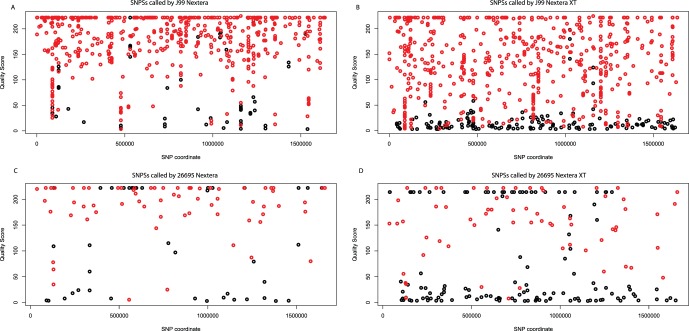
Plots of the SNP quality estimates. Red – Quality score of SNPs called by both Nextera and Nextera XT as the same variant, Black - SNPs called only by the corresponding technology, Nextera (left) and Nextera XT (right).

The mapped data were then compared to the reference genome and differences were determined using samtools [22]. The differences and cross-comparisons are summarised in Table 3. In J99 the Nextera based libraries identified 70 high quality indels and 621 SNPs. The Nextera XT technology identified 59 high quality indels and 771 SNPs. Forty-eight indels and 567 SNPs were common to both technologies (Table S5 in File S1 (indels) and Table S6 in File S1 (SNPs)) and all SNPs were called as the same variant. Quality scores associated with each SNP were plotted (Figure 5a and b).

**Table 3:** Single nucleotide polymorphisms and indels that are common to each technology.

J99 SNPs/Indels			
	Nextera	Nextera XT	Ion Torrent
extera	621/70 (3)	567/48 (3)	558/56 (33)
Nextera XT		771/59 (2)	534/45 (18)
Ion Torrent			658/917 (44)
26695 SNPs/Indels			
	**Nextera**	**Nextera XT**	**Ion Torrent**
Nextera	115/62 (2)	66/53 (2)	57/44 (31)
Nextera XT		133/61 (2)	51/40 (20)
Ion Torrent			65/610 (36)

The first number for each paired comparison represents the quantity of SNPs and the second represents the quantity of indels (ie SNPs/indels). Bracketed numbers represent the number of indels with more than one consensus sequence.

As performed above, the mapped 26695 data were compared to the reference genome and differences identified. The Nextera data predicted 62 indels and 115 high-quality SNPs and the Nextera XT predicted the presence of 61 indels and 133 high quality SNPs. Fifty-three indels were common between the technologies and only 66 SNPs were common (Table S7 in File S1 (indels) and Table S8 in File S1 (SNPs)).

There were 34 base pairs in the published genome sequence, which were not resolved (Table S3 in File S1). All of these bases were non-covered in the Nextera XT sequence data, however, 27 were resolved by Nextera technology. Each consensus base deduced is detailed in Table S9 in File S1.

### Ion Torrent Data Identifies a Similar Number of SNPs and Significantly More Indels

The central premise of massive parallel sequencing is to acquire adequate depth to infer a consensus at each base pair. Therefore, this permits the generation of a limited number of errors within the sequence data. Despite the increased estimated error rate in the Ion Torrent data, we mapped the reads to each control genome to determine if the sequence depth could meaningfully filter out the errors.

Theoretical coverage for J99 and 26695 were 153-fold and 167-fold, respectively. After mapping the sequence data using tmap [20], we found 718 nc (J99) and 429 nc (26695) were not covered by more than 3 nucleotides of mapped sequence data. The genome co-ordinates for the non-covered regions of each genome are detailed in Table S10 in File S1.

The Ion Torrent sequence data mapped to the J99 genome identified 658 high quality SNPS (Table S11 in File S1) and 917 indels (Table S12 in File S1). The Ion Torrent sequence data mapped to the 26695 genome identified 65 high quality SNPS (Table S13 in File S1) and 610 indels (Table S14 in File S1).

We compared the SNPs called by each sequencing technology. The J99 genome sequenced in this experiment has changed remarkably when compared to the originally published sequence. We compared the predicted differences between technologies and found 558 SNPs were commonly identified using Nextera and Ion Torrent and all were called the same variant (Table S15a in File S1). A direct comparison between Nextera XT and Ion Torrent identified 534 common SNPs (Table S16a in File S1). Due to the significantly greater number of non-covered nucleotides using Nextera XT, only SNPs common to both Nextera and Ion Torrent were used for further analyses.

For the 26695-genome sequence, including the 27 bases, which were unresolved in the original genome sequence, 57 SNPs were identified by both Nextera and Ion Torrent technologies (Table S15b in File S1) and 51 were common between Ion Torrent and Nextera XT (Table S16b in File S1).

**Figure 6:**
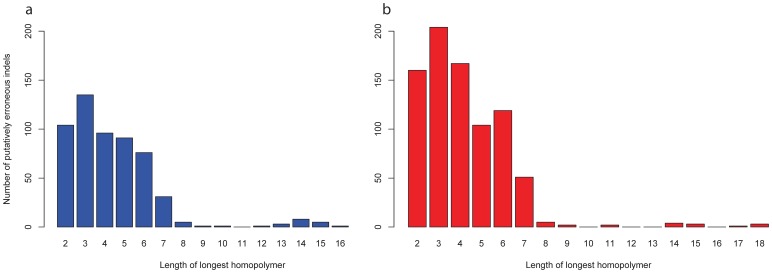
**Homopolymer lengths in the reference sequence at putatively erroneous indel sites determined by Ion Torrent’s PGM.** The *x*-axis represents the longest homopolymer present in the reference genome for each indel site called by the Ion Torrent PGM. The *y*-axis represents the number of homopolymers with at most this length.

The Ion Torrent data predicted 917 and 610 indels in J99 and 26695, respectively, however the Nextera and Nextera XT sequencing approach identified approximately one-tenth this number for each genome. Despite this, there were 56 common indel coordinates in J99, however, 23 were called ambiguously (Table S17a in File S1). A direct comparison between Ion Torrent and Nextera XT yielded 45 common indels (Table S17b in File S1) in J99. A total of 44 common indel co-ordinates were identified in the 26695-genome with 31 called as the same indel and the remainder defined as a possibly more than one variant (Table S18a in File S1). Ion Torrent compared with Nextera XT identified 40 common indels, 20 of which were ambiguously defined (Table S18b in File S1). Due to previous literature detailing the Ion Torrent PGM’s limitations in its ability to determine the exact composition of homopolymeric tracts [7], we analysed the nucleotide composition of indels which were specific to the Ion Torrent data (858 in J99, 567 in 26695). Homopolymers of length greater than or equal to 2 were present in 825 indels in J99 and 567 indels in 26695 and their composition is represented in Figure 6.

### Coding Sequence, de novo Assemblies of Nextera Data are more Accurate than Nextera XT and Ion Torrent

Mapping sequence data to determine strain-to-strain differences is effective for highly clonal species [23], [24], however, to elucidate an accurate genome sequence from a novel strain of a highly variable species such as *H. pylori,* it requires the data are assembled *de novo.* This method identifies identical *k-*mers between sequence reads and attempts to rebuild the entire genome. We assembled each dataset using the Velvet algorithm [8] for Illumina data or Mira [21] for the Ion Torrent data. The fewest contigs were obtained by setting the Velvet *k*-mer value to 71 (for a summary of assembly statistics see Table S19 in File S1).

The reference genomes were updated according to the consensus SNPs and indels identified by both sequencing technologies. Ambiguous calls were ignored. Bases identified as having changed in one technology but not the other were excluded from further analyses of coding sequences. Therefore, we analysed 1419 of 1488 predicted coding sequences in J99 and 1493 of 1573 predicted coding sequences in 26695. The accuracy of the Nextera assemblies was consistently high compared to the Nextera XT and Ion Torrent assemblies (Table 4).

**Table 4:** Global analysis of coding sequence accuracy of each assembly.

Genome	Ion Torrent		Nextera		Nextera XT	
	Correct CDS/Total CDS*	Percentage	Correct CDS/Total CDS*	Percentage	Correct CDS/Total CDS*	Percentage
J99	1149/1419	81.0%	1353/1419	95.3%	981/1419	69.1%
26695	1014/1493	67.9%	1430/1493	95.8%	1165/1493	78.0%

* Represents the number assembled correctly/Expected number to be assembled correctly; CDS, coding sequence.

### De novo MLST Reconstruction is Inconsistent across Sequencing Technologies and Genomes

To determine the usefulness of the assemblies for characterising strains according to the established method of multi-locus sequence typing (MLST), we extracted the MLST from the assembled data and compared these to the data from the *Helicobacter* database at *Pub*MLST (Table 5). We also confirmed sequence changes in the *mutY* and *atpA* sequence of these genes using standard Sanger sequencing technology.

**Table 5:** Percentage sequence identity of MLST alleles extracted from assembled data.

	J99 Genome				26695 Genome			
MLST Allele	Expected MLST allele number	Ion Torrent	Nextera	Nextera XT	Expected MLST allele number	Ion Torrent	Nextera	Nextera XT
*atpA*	199*	99.84	NP	NP	181	100	100	100
*efp*	199	100	100	100	181	100	100	100
*mutY*	199*	99.76	99.76	99.76	181*	99.76	99.76	99.76
*ppa*	199	100	100	100	181	100	100	100
*trpC*	199	100	100	100	181	100	100	100
*ureI*	199	100	100	100	181	100	100	100
*yphC*	199	100	100	100	181	100	100	100
*vacA*	199*	99.77	99.77	99.77	181	100	100	100

* template changed from original submission to *Pub*MLST database and confirmed by Sanger sequencing. NP = not present.

Interestingly, the Ion Torrent data assemblies encoding the MLST genes for both J99 and 26695 were accurately assembled using the mira algorithm, despite the poorer overall accuracy of all coding sequences. Conversely, the Nextera and Nextera XT were not completely accurate. The *atpA* gene was missing from the final assembly of J99 using both Nextera and Nextera XT.

### The cagPAI is Difficult to Assemble de novo for All Technologies

To determine if accurate *de novo* assembly of the *cagPAI* was possible using these technologies, we analysed the sequence identity of the *cagPAI* genes. For J99, the *cag*PAI sequences were found in three different contigs for the Ion Torrent and Nextera data and in 20 contigs of the Nextera XT assembly. The J99 Nextera XT *cag*PAI data were not analysed further due to sheer number of contigs the *cag*PAI sequences were found in. After correcting for template changes in *cagA* (jhp_0495, 1 SNP) and *cagH* (jhp_0489, 3 SNPs) the accuracy of the assemblies was lower in the Ion Torrent assembly (22 of 27 were 100% accurate) compared with the Nextera (26 of 27 genes were 100% identical) (Table 6).

**Table 6:** Analysis of the accuracy of the assembled *cagPAI* coding sequences.

	J99					26695				
		Ion Torrent		Nextera			Ion Torrent		Nextera	
Gene Name	Gene ID	Contig(s)	Percentage Identity	Contig(s)	Percentage Identity	Gene ID	Contig(s)	Percentage Identity	Contig	Percentage Identity
*cagζ*	jhp_0469	c42	100	22	100	HP0520	c40	100	23	100
*cagε*	jhp_0470	c42	100	22	100	HP0521+	–	–	–	–
*cagδ*	jhp_0471	c42	100	22	100	HP0522	c40	100	23	100
*cagγ*	jhp_0472	c42	100	22	100	HP0523	c40	100	23	100
*cagβ*	jhp_0473	c42	99.69	22	100	HP0524	c40/c188	–	23	100
*cagα*	jhp_0474	c42	99.80	22	100	HP0525+	–	–	–	–
*cagZ*	jhp_0475	c42	100	22	100	HP0526	c188	100	23	100
*cagY*	jhp_0476	c42/c44	–	22/36/53	–	HP0527*	c130/c188	–	23/31/33/28	–
*cagX*	jhp_0477	c44	100	53	100	HP0528	c130	99.87?	23	100
*cagW*	jhp_0478	c44	100	53	100	HP0529	c130	99.94?	23	100
*cagV*	jhp_0479	c44	100	53	100	HP0530	c130	100	23	100
*cagU*	jhp_0480	c44	100	53	100	HP0531	c130	100	23	100
*cagT*	jhp_0481	c44	100	53	100	HP0532	c130	100	23	100
*cagS*	jhp_0482	c44	100	53	100	HP0534	c130	100	23	100
*cagQ*	jhp_0483	c44	100	53	100	HP0535	c130	100	23	100
*cagP*	jhp_0484	c44	100	53	100	HP0536	c130	100	23	100
*cagM*	jhp_0485	c44	99.91	53	100	HP0537	c130	99.91?	23	100
*cagN*	jhp_0486	c44	100	53	100	HP0538	c130	99.89?	23	100
*cagL*	jhp_0487	c44	100	53	100	HP0539	c130	100	23	100
*cagI*	jhp_0488	c44	100	53	100	HP0540	c130	100	23	100
*cagH*	jhp_0489*	c44	99.73	53	99.73	HP0541*	c130	99.82	23	99.82
*cagG*	jhp_0490	c44	100	53	100	HP0542	c70/c130	–	23	100
*cagF*	jhp_0491	c44	100	53	100	HP0543*	c70	99.75?	23	99.88
*cagE*	jhp_0492	c14/c44	–	53	100	HP0544	c70	100	23	100
*cagD*	jhp_0493	c14	100	53	100	HP0545	c70	99.84?	23	100
*cagC*	jhp_0494	c14	100	53	100	HP0546	c70	100	23	100
*cagA*	jhp_0495*	c14	99.97	53	99.97	HP0547*	c70	99.92?	23	99.97

* changes in the template that have occurred since the data were submitted to NCBI,

?error due to indel,+(plus) text represents a pseudogene, – (dash) no comparative analysis performed.

For 26695, we performed the same analysis and found the *cag*PAI sequence data present mostly on a single contig with remnants of the repetitive gene *cagY*, found on the same contig and 3 further contigs. Of the 25 coding sequences in the *cag*PAI of 26695, only 1 (*cagY*, HP_0527) was assembled incorrectly in the Nextera assembly compared to 7 in the Ion Torrent assembly (Table 6).

## Discussion

High-throughput sequencing technologies have been used to infer strain-to-strain relationships in many bacterial species, however, many of these studies have focused on a single sequence type (ST) or clonal species. *H. pylori* is fascinating in its allelic diversity with 1456 unique MLST combinations in 1551 analysed isolates. Mapping-based strategies for high-throughput sequence data are severely limited by allelic diversity, as mapping algorithms generally permit only a few differences within the first portion of each read. *De novo* assembly is theoretically more appropriate for analysing sequence data derived from *H. pylori* isolates, however, the low GC nature of the genome and large numbers of repetitive regions may affect the accuracy. We have analysed the coding sequence accuracy of the assemblies after rigorously removing all putative template changes in the reference genomes J99 and 26695. Overall, more genes (∼95%) were assembled correctly using the Illumina Nextera sequencing technology with variable results from the Nextera XT and Ion Torrent methods. Despite this significant difference in accuracies, we found that, in the two genomes, we could not consistently extract the MLST type using Illumina-based methods from *de novo* assemblies, with the *atpA* gene missing from both Nextera and Nextera XT J99 assemblies. Our analysis used the freely available software Velvet assembler, however, if the Nextera data are assembled using the proprietary CLC bio genomic benchwork *de novo* assembler (Ver. 6.0.2), the *atpA* gene is accurately assembled (data not shown). This greater accuracy of the CLC assembler has been noted previously [7] but we chose to focus on lower cost methodologies. This lack of the *atpA* gene in the assembly is solely to due to the assembler as the data are clearly present in the raw sequence. We showed that the Ion Torrent assemblies were accurate in reconstructing the MLST for both genomes, yet, for clinically important, virulence-associated loci *cag*PAI, the Nextera system is more accurate and incorporated fewer indels. It is important to note that since we performed the Ion Torrent sequencing, a newer version of the base calling software has been released. This more recent version reportedly improves the accuracy of homopolymeric tract composition. It is evident that the Ion Torrent data suffers dramatically by the inability to correctly determine homopolymeric tracts, which may or may not be improved by the updated software. Mapping the Ion Torrent data identified approximately 10-fold more indels than the Illumina derived data and these have clearly been incorporated into the *de novo* assemblies reducing the accuracy of the coding sequences.

When directly comparing the Nextera and Nextera XT methodologies the estimated coverages are relatively similar, however, significantly more nucleotides are covered by the Nextera method. The quantity of starting material is different between the two protocols requiring 50 ng for Nextera and 1 ng for Nextera XT. It is tempting to hypothesise that the difference in this amount leads to the increase in non-covered regions and it is the amplification step that provides for the similar number of reads. However, this assumption is further complicated by the fact that the unique and mapped, or low covered 31 mers have a differing average GC between the technologies. Further investigation is warranted as to whether this difference in coverage is due to the quantity of input genomic DNA, due to the enzymatic digestion, the different amount of PCR amplification or an unknown mechanism of this fledgling protocol.

The analysis of our data shows that with the current benchtop sequencing technologies, despite the fact that Ion Torrent is proficient at assembling the MLST data, the preferred methodology should be Illumina MiSeq Nextera, which exhibited greater overall accuracy of *de novo* assemblies. If using this method to infer MLST-based phylogenetic relationships, and, if information specific to the *atpA* gene is not present, it could be extracted from the raw sequence data using mapping technology and the complete set of *atpA* alleles.

## Supporting Information

Figure S1 **QRQC analysis of 26695 raw sequence data derived by Ion Torrent.** (a) Plot of the quality distribution by base position. (b) Histogram of the read lengths. (c) Proportions of each base with respect to the position in the read.
(EPS)

Figure S2 **QRQC analysis of J99 raw sequence data derived by Illumina Nextera.** Plots of the quality distribution by base position for read 1 (a) and read 2 (b). Proportions of each base with respect to the position in read 1 (c) and read 2 (d). Histograms of the read 1 lengths (e) and read 2 lengths (f). Mean GC proportion for each position in read 1 (g) and read 2 (h).
(EPS)

Figure S3 **QRQC analysis of J99 raw sequence data derived by Illumina Nextera XT.** Plots of the quality distribution by base position for read 1 (a) and read 2 (b). Proportions of each base with respect to the position in read 1 (c) and read 2 (d). Histograms of the read 1 lengths (e) and read 2 lengths (f). Mean GC proportion for each position in read 1 (g) and read 2 (h).
(EPS)

Figure S4 **GC content analysis of unique 31-mers.** *Modified* Boxplots of the GC content for all unique 31-mers that map to the reference genomes. (a) represents a subset of the Ion Torrent data (b) represents a subset of the Nextera data and (c) a subset of the Nextera XT data. The dark horizontal line represents the median, upper and lower limit of the box represents 1.5 times the interquartile range, the whiskers represent the largest or smallest non-outlier values and circles represent putative outliers.
(EPS)

File S1 **Compressed file containing Tables S1–S19.**
(ZIP)
